# Role of dimensionality in complex networks

**DOI:** 10.1038/srep27992

**Published:** 2016-06-20

**Authors:** Samuraí Brito, L. R. da Silva, Constantino Tsallis

**Affiliations:** 1 Universidade Federal do Rio Grande do Norte, Departamento de Física Teórica e Experimental, Natal-RN, 59078-900, Brazil; 2 National Institute of Science and Technology of Complex Systems, Brazil; 3 Centro Brasileiro de Pesquisas Físicas, Rua Xavier Sigaud 150, 22290-180 Rio de Janeiro-RJ, Brazil, and Santa Fe Institute, 1399 Hyde Park Road, New Mexico 87501, USA

## Abstract

Deep connections are known to exist between scale-free networks and non-Gibbsian statistics. For example, typical degree distributions at the thermodynamical limit are of the form 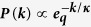, where the *q*-exponential form 
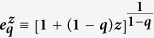
 optimizes the nonadditive entropy *S*_*q*_ (which, for *q* → 1, recovers the Boltzmann-Gibbs entropy). We introduce and study here *d*-dimensional geographically-located networks which grow with preferential attachment involving Euclidean distances through 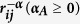. Revealing the connection with *q*-statistics, we numerically verify (for *d* = 1, 2, 3 and 4) that the *q*-exponential degree distributions exhibit, for both *q* and *k*, universal dependences on the ratio *α*_*A*_/*d*. Moreover, the *q* = 1 limit is rapidly achieved by increasing *α*_*A*_/*d* to infinity.

Networks emerge spontaneously in many natural, artificial and social systems. Their study is potentially important for physics, biology, economics, social sciences, among other areas. For example, many empirical studies have identified peculiar properties in very different networks such as the Internet and online social networks (e.g., Facebook), citations networks, neurons networks^1^,^2^,^3^, to quote but a few. An ubiquitous class of such networks is constituted by the scale-free ones (more precisely, asymptotically scale-free). As we shall soon verify, these networks can be seen as a particular application of nonextensive statistical mechanics, based on the nonadditive entropy 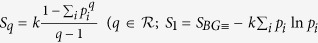, where *BG* stands for *Boltzmann-Gibbs*)^4^,^5^,^6^. This current generalization of the BG entropy and corresponding statistical mechanics has been widely successful in clarifying the foundations of thermal statistics as well as for applications in complex systems in high-energy collisions at LHC/CERN (CMS, ALICE, ATLAS and LHCb detectors) and at RHIC/Brookhaven (PHENIX detector)^7^,^8^,^9^,^10^,^11^,^12^,^13^,^14^,^15^,^16^, cold atoms^17^, dusty plasmas^18^, spin-glasses^19^, trapped ions^20^, astrophysical plasma^21^,^22^, biological systems^23^, type-II superconductors^24^, granular matter^25^, the Kuramoto model at the edge of chaos^26^, low-dimensional maps, for instance the (area-preserving) standard map^27^ (see Bibliography in http://tsallis.cat.cbpf.br/biblio.htm). Many other physical situations are described which are analogous, such as long-range-interacting Hamiltonians, for example, gravitational problems like globular clusters, spins systems, like the Ising, XY and Heisenberg long-range models. We may also point out random-walk anomalous diffusion where the jumps obey a power low probability distribution function. Some (naturally not all) of the properties of long-range-interacting systems may be described as forming complex network where the sites are linked according to power-law preferential attachment. In the present work we address a wide class of this kind of problems focusing on some basic universality relations.

The deep relationship between scale-free networks and *q*-statistics started being explored in 2005^28^,^29^,^30^, and is presently very active^31^,^32^,^33^,^34^,^35^. The basic connection comes (along the lines of the BG canonical ensemble) from the fact that, if we optimize the functional 
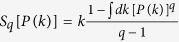
 with the constraint 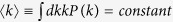 or analogous (*k* being the degree of a generic site, i.e., the number of links connected to a given site; *P*(*k*) denotes the degree or connectivity distribution), we straightforwardly obtain 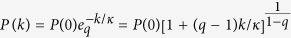, which turns out to be the generic degree distribution for virtually all kinds of scale-free networks. The *q*-exponential function is defined as 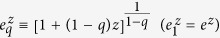. We verify that, for *q* > 1 and *k* → ∞, *P*(*k*) ~ 1/*k*^*γ*^ with *γ* ≡ 1/(*q* − 1). The classical result *γ* = 3^36^ corresponds to *q* = 4/3.

In the present work we address the question of how universal such results might be, and more specifically, how *P*(*k*) varies with the dimension *d* of the system?

Our growing model starts with one site at the origin. We then stochastically locate a second site (and then a third, a fourth, and so on up to *N*) through the *d*-dimensional isotropic distribution


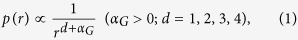


where *r* ≥ 1 is the Euclidean distance from the newly arrived site to the center of mass of the pre-existing system (in one dimension, *r* = |*x*|; in two dimensions, 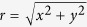; in three dimensions 
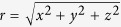
, and so on); we assume angular isotropy; *p*(*r*) is zero for 0 ≤ *r* < 1; the subindex *G* stands for *growth*. We consider *α*_*G*_ > 0 so that the distribution *P*(*r*) is normalizable; indeed, 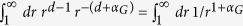, which is finite for *α*_*G*_ > 0, and diverges otherwise. See Fig. 1.

Every new site which arrives is then attached to one and only one site of the pre-existing cluster. The choice of the site to be linked with is done through the following preferential attachment probability:


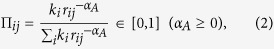


where *k*_*i*_ is the connectivity of the *i*-th pre-existing site (i.e., the number of sites that are already attached to site *i*), and *r*_*ij*_ is the Euclidean distance from site *i* to the newly arrived site *j*; subindex *A* stands for *attachment*.

For *α*_*A*_ approaching zero and arbitrary *d*, the physical distances gradually loose relevance and, at the limit *α*_*A*_ = 0, all distances becomes irrelevant in what concerns the connectivity distribution, and we therefore recover the Barabási-Albert (BA) model^36^, which has topology but no metrics. The BA model was extended^37^ in such a way that it would be able to yield an exponent *γ* such that 2 < *γ* < 3, thus making the model more realistic. In this work they showed a topological phase transition which range from scale-free networks to exponential networks through three control parameters (addition of links, redirection of edges, and addition of new sites). In the present paper we show that, for arbitrary dimensionality, *γ* can be controlled in a kind of simpler manner, namely by metric changes through only one control parameter (namely the ratio *α*_*A*_/*d*) in the structure of the network. Notice, however, that the BA generalized model is not a particular case of our model, and neither the other way around.

Large-scale simulations have been performed for the (*d* = 1, 2, 3, 4) models for fixed (*α*_*G*_, *α*_*A*_), and we have verified in all cases that the degree distribution *P*(*k*) is completely independent from *α*_*G*_: see Fig. 2. Using this fact, we have arbitrarily fixed *α*_*G*_ = 2, and have numerically studied the influence of (*d*, *α*_*A*_) on *P*(*k*): see Figs 3 and 4. In all cases, the *q*-exponential fittings 
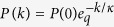
 with *q* > 1 and *κ* > 0 have been remarkably good. To test the goodness of fit, we performed Kolmogorov-Smirnov test^38^ (see Table 1). To deal with the problem that the data are very sparse in the tail, we excluded data points with sample probability less than 10^−6^. The best fitting values for (*q*, *κ*) are indicated in Fig. 5. From normalization of *P*(*k*), *P*(0) can be expressed as a straightforward function of (*q*, *κ*).

Our most remarkable results are presented in Fig. 6, namely the fact that both the index *q* and the characteristic degree (or “effective temperature”) *κ* do not depend from (*α*_*A*_, *d*) in an independent manner but *only from the ratio α*_*A*_/*d*. This nontrivial fact puts the growing *d*-dimensional geographically located models that have been introduced here for scale-free networks, on similar footing as long-range-interacting many-body classical Hamiltonian systems such as the inertial XY planar rotators^39^,^40^,^41^,^42^ (possibly the generic inertial *n*-vector rotators as well^43^,^44^) and Fermi-Pasta-Ulam oscillators, assuming that the strength of the two-body interaction decreases with distance as 1/(*distance*)^*α*^. Moreover, as first pointed out generically by Gibbs himself^45^, we have the facts that the BG canonical partition function of these classical systems anomalously diverges with size for 0 ≤ *α*/*d* ≤ 1 (long-range interactions, e.g., gravitational and dipole-monopole interactions) and converges for *α*/*d* > 1 (short-range interactions, e.g., Lennard-Jones interaction), and the internal energy per particle is, in the thermodynamical limit, constant for short-range interactions whereas it diverges like *N*^1−*α*/*d*^ for long-range interactions, *N* being the total number of particles.

If all these meaningful scalings are put together, we obtain a highly plausible scenario for the respective domains of validity of the Boltzmann-Gibbs (additive) entropy and associated statistical mechanics, and that of the nonadditive entropies *S*_*q*_ (with *q* ≠ 1) and associated statistical mechanics.

Finally, we notice in Fig. 6 that both *q* and *κ* approach quickly their BG limits (*q* = 1) for *α*_*A*_/*d* → ∞. Moreover, the same exponential *e*^1−*α*/*d*^ appears in both heuristic expressions for *q* and *κ*. Consequently, the following *linear* relation can be straightforwardly established:


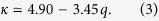


In fact, this simple relation is numerically quite well satisfied as can be seen in Fig. 7. Its existence reveals an interesting peculiarity of the nature of *q*-statistics. If in the celebrated BG factor *e*^−*energy*/*kT*^, corresponding to *q* = 1, we are free to consider an arbitrary value for *T*, how come in the present problem, *κ* is not a free parameter but has instead a fixed value for each specific model that we are focusing on? This is precisely what occurs in the high-energy applications of *q*-statistics, e.g., in quark-gluon soup^46^ where *q* = 1.114 and *T* = 135.2 *Mev*, as well as in all the LHC/CERN and RHIC/Brookhaven experiments^7^. Another example which is reminiscent of this type of behavior is the sensitivity to the initial conditions at the edge of chaos (Feigenbaum point) of the logistic map; indeed, the inverse *q*-generalized Lyapunov exponent satisfies the linear relation 1/*λ*_*q*_ = 1 − *q*^47^,^48^. The cause of this interesting and ubiquitous feature comes from the fact that *q*-statistics typically emerges at critical-like regimes and is deeply related to an hierarchical occupation of phase space (or Hilbert space or Fock space), which in turn points towards asymptotic power-laws (see also^49^). In other words, *κ* plays a role analogous to a critical temperature, which is of course not a free parameter but is instead fixed by the specific model.

## Additional Information

**How to cite this article**: Brito, S. *et al*. Role of dimensionality in complex networks. *Sci. Rep.* **6**, 27992; doi: 10.1038/srep27992 (2016).

## Figures and Tables

**Figure 1:**
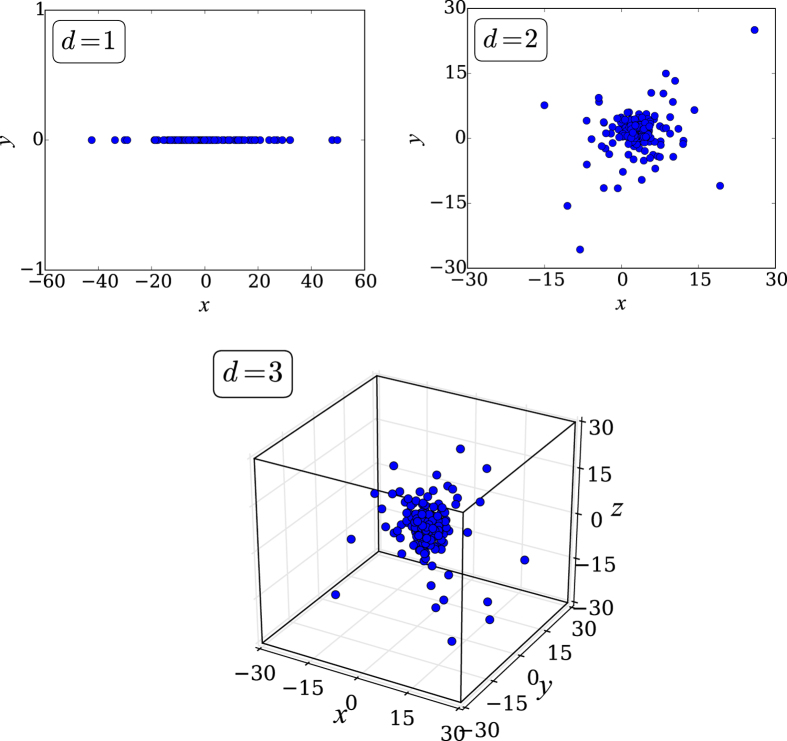
Distribution of *N* = 500 sites obtained with Eq. (1) for *α*_*A*_ = 2.0, *α*_*G*_ = 0.0, and *d* = 1, 2, 3.

**Figure 2:**
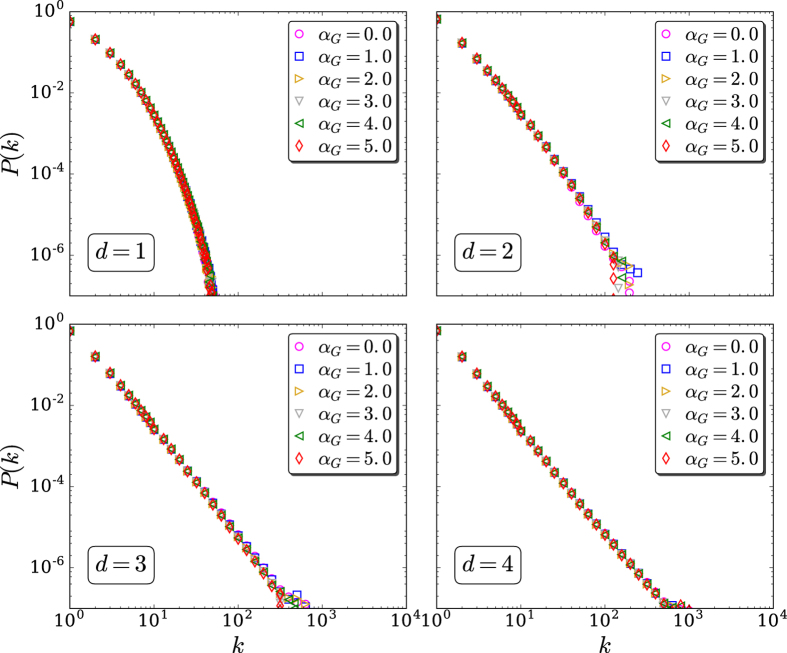
Connectivity distribution for *d* = 1, 2, 3, 4, *α*_*A*_ = 2.0 and typical values for *α*_*G*_. The simulations have been run for 10^3^ samples of *N* = 10^5^ sites each. We verify that *P*(*k*) is independent from *α*_*G*_ (∀*d*). Logarithmic binning was used whenever convenient.

**Figure 3:**
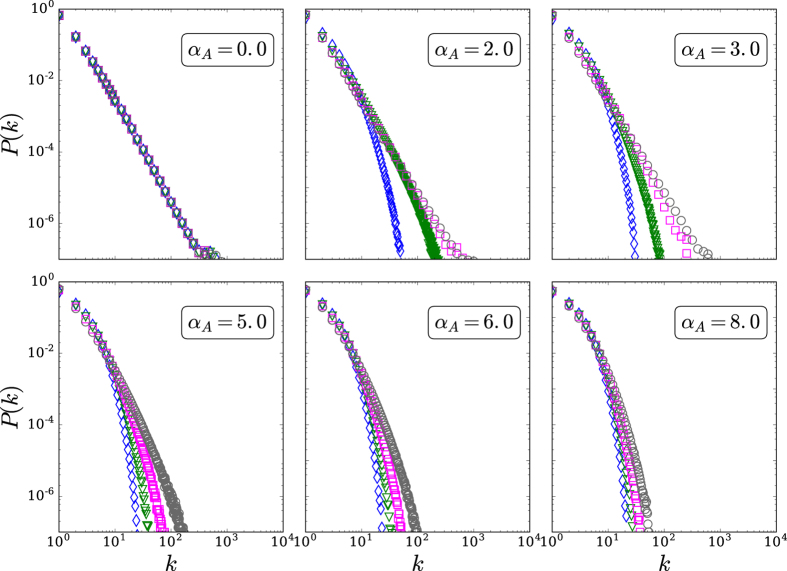
Degree distribution for d = 1(*blue diamonds*), 2(*green triangles*), 3(*magenta squares*), 4(*grey circles*), and typical values of *α*_*A*_, with *α*_*G*_ = 2.0. The simulations have been run for 10^3^ samples of *N* = 10^5^ sites each. Logarithmic binning was used whenever convenient.

**Figure 4:**
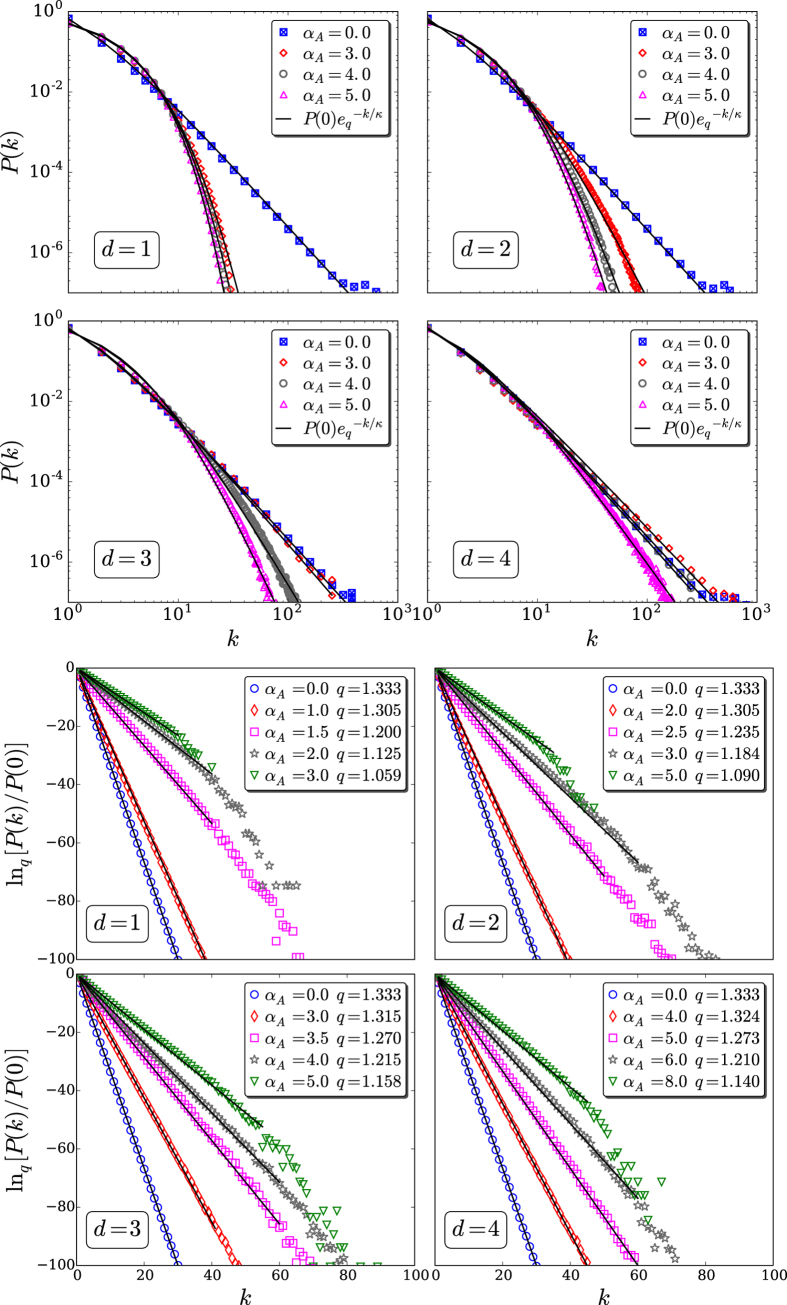
Fittings of the *d* = 1, 2, 3, 4 connectivity distributions with the function 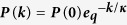, where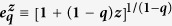. The data are those of Fig. 3. *Top:* log-log representation. *Bottom:* ln_*q*_[*P*(*k*)/*P*(0)] versus *k* representation. Notice that straight lines in a ln_*q*_–linear representation univocally determine the *q*-exponential function. The fitting parameters are exhibited in Fig. 5. The numerical failure, at large enough values of *k*, with regard to straight lines are finite-size effects that gradually disappear when we approach the thermodynamic limit *N* → ∞. Logarithmic binning was used whenever convenient.

**Figure 5:**
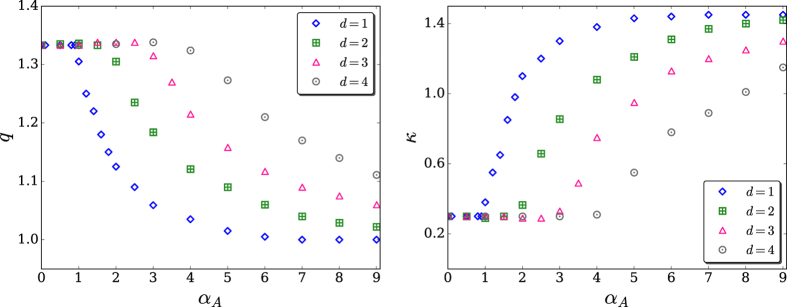
*q* and *κ* for *d* = 1, 2, 3, 4. For *α*_*A*_ = 0 and ∀*d*, we recover the Barabási-Albert universality class *q* = 4/3 (hence *γ* = 3)^36^, which has no metrics.

**Figure 6:**
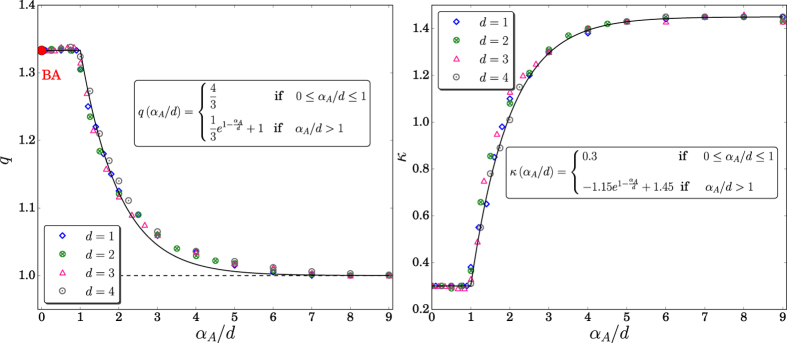
*q* and *κ* versus *α*_*A*_/*d* (same data as in Fig. 5). We see that *q* = 4/3 for 0 ≤ *α*_*A*_/*d* ≤ 1, and a nearly exponential behavior emerges for *α*_*A*_/*d* > 1 (∀*d*); similarly for *κ*. These results exhibit the universality of both *q* and *κ*. The red dot indicates the Barabási-Albert (BA) universality class *q* = 4/3^36^. In what concerns the universal *q* = 4/3 cut-off (i.e., the 1/(*q* − 1) = 3 cut-off), see^50^ and references therein.

**Figure 7:**
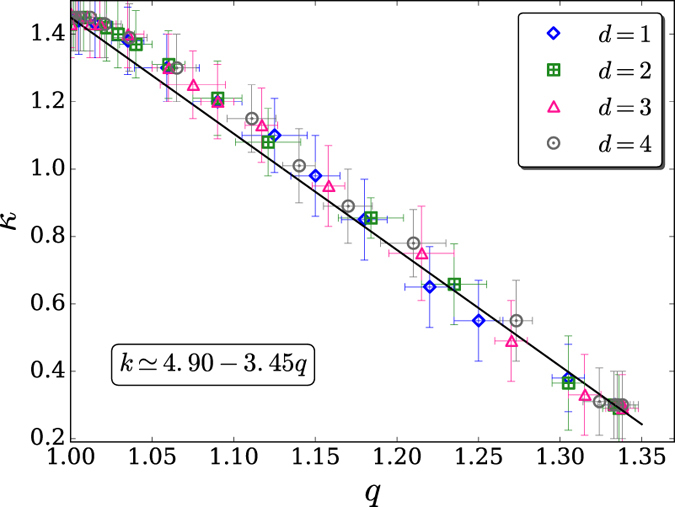
All the values of *q* and *κ* for the present *d* = 1, 2, 3, 4 models follow closely the linear relationEq. (3) (continuous straight line). The upmost value of *q* is 4/3, yielding 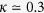 (∀*d*).

**Table 1:** Some *q* and *κ* parameters from the Fig. 4 for the best fit to a *q*-exponential function for *d* = 1, 2, 3, 4 connectivity distributions.

Dimension	*α*_*A*_	*q*	*κ*	*p*
*d* = 1	0.0	1.333 ± 0.01	0.30 ± 0.10	0.98
	1.0	1.305 ± 0.01	0.38 ± 0.10	1.00
	1.5	1.200 ± 0.02	0.75 ± 0.13	1.00
	2.0	1.125 ± 0.02	1.10 ± 0.11	0.84
	3.0	1.059 ± 0.01	1.30 ± 0.10	0.99
*d* = 2	0.0	1.333 ± 0.01	0.30 ± 0.10	0.99
	2.0	1.305 ± 0.01	0.36 ± 0.14	0.94
	2.5	1.235 ± 0.02	0.66 ± 0.12	0.96
	3.0	1.184 ± 0.02	0.85 ± 0.06	0.96
	5.0	1.090 ± 0.01	1.21 ± 0.11	0.93
*d* = 3	0.0	1.333 ± 0.01	0.30 ± 0.10	0.98
	3.0	1.315 ± 0.01	0.33 ± 0.12	0.94
	3.5	1.270 ± 0.01	0.49 ± 0.12	0.99
	4.0	1.215 ± 0.02	0.75 ± 0.14	0.99
	5.0	1.158 ± 0.01	0.95 ± 0.12	0.88
*d* = 4	0.0	1.333 ± 0.01	0.30 ± 0.10	0.96
	4.0	1.324 ± 0.01	0.31 ± 0.10	0.99
	5.0	1.273 ± 0.01	0.55 ± 0.12	0.73
	6.0	1.210 ± 0.02	0.78 ± 0.10	0.79
	8.0	1.140 ± 0.01	1.01 ± 0.11	1.00

The first two columns are the parameters of the network. The next two columns are the fit parameters to the *q*-exponential. The last column corresponds to the *p*-values for nonparametric statistical Kolmogorov-Smirnov test. In this test fitting curves are considered acceptable if *p* > 0.05. We can see in our results *p* ≥ 0.73 indicating that connectivity distributions are very well described for *q*-exponential functions. We analyzed networks of the 10^5^ sites and 10^3^ samples. The errors that are indicated were calculated through upper bounds of those obtained by using the chi-square method.
